# Sweet Clover (*Melilotus* spp.) as a Source of Biologically Active Compounds

**DOI:** 10.3390/molecules30030526

**Published:** 2025-01-24

**Authors:** Patrycja Sowa-Borowiec, Maria Czernicka, Wacław Jarecki, Małgorzata Dżugan

**Affiliations:** 1 Department of General and Inorganic Chemistry, Faculty of Chemical Engineering and Technology, Cracow University of Technology, 31-155 Cracow, Poland; 2 Department of Bioenergetics, Food Analysis and Microbiology, University of Rzeszow, 35-601 Rzeszow, Poland; mczernicka@ur.edu.pl; 3 Department of Crop Production, University of Rzeszów, Zelwerowicza 4, 35-601 Rzeszow, Poland; 4 Department of Chemistry and Food Toxicology, University of Rzeszow, Ćwiklińskiej 1a, 35-601 Rzeszow, Poland

**Keywords:** biologically active compounds, antioxidant activity, *Melilotus* spp., phenolic compounds, *Melilotus albus*, *Melilotus officinalis*, coumarin

## Abstract

Sweet clover, particularly white sweet clover (*Melilotus albus*), is an underexplored plant that has gained attention in recent years. This study compared the bioactive compounds content in the flowers, leaves, and stems of *Melilotus albus* Medic. to the well-known health-promoting *Melilotus officinalis* (L.) Lam. Both fresh and dried plant materials were analysed, with chromatographic assessments preceded by the optimisation of the extraction method (using 50% aqueous ethanol–water and the plant sample ground in a mortar, followed by 30 min of ultrasound-assisted extraction). Flower and leaf extracts were also evaluated for their total phenolic content (TPC) and antioxidant activity using FRAP and DPPH**·** assays. Both plant species were rich in phenolic compounds, including coumarins, phenolic acids, and flavonoids. HPLC-PDA analysis revealed similar profiles for both species, with quantitative differences in individual compound fractions. The highest coumarin content was found in the fresh flowers, followed by the leaves and stems. No significant species-specific differences in the coumarin content were observed. However, the flowers of *M. albus* were a richer source of flavonoids, with the highest hyperoside content. The flavonoid profile in the leaf extracts was similar to that of the flowers, but the content was about four times lower in the leaves and even lower than in the stems. Interestingly, the leaf extracts exhibited higher antioxidant activity than the flower extracts. The presented findings suggest that white sweet clover is an equally valuable source of health-promoting compounds as yellow sweet clover.

## 1. Introduction

Sweet clover (*Melilotus* spp.) is a forage plant belonging to the Fabaceae family (legumes) and the subfamily Faboideae. It is classified within the *Trifolieae* tribe, alongside clover (*Trifolium*), alfalfa (*Medicago*), and fenugreek (*Trigonella*) [1]. The genus name *Melilotus* is derived from the Greek words *meli* (méli, μέλι), meaning honey, and *lotos* (lôtós, λωτός), referring to clover (due to the morphological similarity between sweet clover leaves and those of clover) or fodder (as described by Homer as feed for horses) [2,3]. There are approximately 25 species of sweet clover, with the most widespread being white sweet clover (*Melilotus albus* Medic.) and yellow sweet clover (*Melilotus officinalis* (L.) Lam.) [2,4]. These species can occur in both biennial and annual forms [4]. Sweet clover is a plant of Eurasian origin; however, it is now distributed across nearly all the regions of the world [1]. White sweet clover reaches a height of 1.5 to 2 m, whereas yellow sweet clover is shorter (approximately 1 m tall). Annual forms of *Melilotus* are generally smaller, with thinner stems and a reduced height. The leaves of sweet clover are pinnately trifoliate, with finely toothed leaflets and stipules fused to the petiole. Its flowers are small, yellow or white (depending on the species), and arranged in relatively loose, elongated racemes. It produces many small, ovoid seeds enclosed in pods that are initially greenish or yellowish-brown and quickly darken to brown [5].

The health-promoting properties and chemical composition of *Melilotus* species, with the exception of *Melilotus officinalis*, remain poorly understood. In recent years, studies have been conducted to identify molecular markers enabling the differentiation of individual *Melilotus* species. Researchers have analysed the genetic variability of sweet clovers and constructed phylogenetic trees, providing insights into their interrelationships and genetic affinities with other species, for example, based on chloroplast genome analysis [6,7,8,9,10,11].

In traditional herbal medicine, sweet clover (*Melilotus*) is used as an infusion to treat various conditions, including digestive issues, conjunctivitis, arthritis, bronchitis, haemorrhoids, stomach ulcers, and an oedema of the lower limbs due to poor circulation, as well as for external application on wounds, burns, and boils [2,5]. According to the European Pharmacopoeia, the herbal raw material referred to as *Meliloti* herba consists of the whole or cut dried aerial parts of *Melilotus officinalis* (L.) Lam., with a minimum coumarin content of 0.3% [12]. Therefore, the majority of studies on the health-promoting properties of sweet clovers have focused on this species. Very good results have been achieved in the treatment of diabetic foot ulcer wounds using the commercially available dry extract of yellow sweet clover, Semelil (ANGIPARSTM), as demonstrated in several clinical studies [2]. In the studies conducted by Derakhshan et al. [13], three-layered PCL–collagen nanofibres containing *M. officinalis* extract were tested for diabetic ulcer healing in a rat model. Based on histopathological and histomorphometric evaluations, dressings enriched with the plant extract (particularly those containing 0.08 g of the extract) were shown to be promising in promoting the healing of diabetic ulcers [13]. Similarly, favourable outcomes were reported in the treatment of third-degree burns in rats using *M. officinalis* gels. They reduced swelling and the area of tissue necrosis compared to the control group [14]. Safarpour et al. [15] analysed the antioxidant and anti-inflammatory effects of the gel (10 and 20%) and aqueous extract (orally, 500 and 1000 mg/kg) of yellow sweet clover in a Rattus norvegicus model with induced ulcerative colitis. After seven days, colon tissues were examined for macroscopic and histopathological changes, as well as oxidative stress. Significant differences were observed regarding pathological changes, malondialdehyde levels, and weight improvements compared to the untreated control group. In the study by Zhao et al. [16], it was demonstrated that aqueous extracts (100, 250, and 500 mg/kg, administered for three days) improved apoptosis in the brain tissues of rats with cerebral ischemia by reducing cerebral thrombosis, oxidative stress, and inflammatory mediators. Bazazzadegan et al. [17] found that *M. officinalis* extract (an intraperitoneal injection at the dose of 20 mg/kg/day for 21 days) may significantly influence the expression of genes associated with Alzheimer’s disease. Moreover, *M. officinalis* has been applied in the treatment of lymphoedema as well as oedema caused by a chronic venous insufficiency. In a clinical study, patients with a chronic venous insufficiency were treated with 200 mg of dry *M. officinalis* herb extract daily for 15 days. The treatment effectively reduced ankle swelling, nighttime calf cramps, and the sensation of heavy legs. Meanwhile, patients with lower limb lymphoedema received 400 mg/day of the herb extract for six months in conjunction with compression therapy. Significant improvement was observed after treatment [18]. Numerous in vitro and in vivo studies have shown that sweet clover extract exhibits anti-inflammatory, anticoagulant, anticancer, antibacterial, antioxidant, and antihypertensive properties [19,20,21,22,23,24,25,26,27,28]. It is believed that the compound responsible for most of the health-promoting properties of sweet clover is coumarin. Coumarin (1,2-benzopyrone, 2H-1-benzopyran-2-one, cis-*o*-coumaric acid lactone) is also responsible for its distinctive sweet fragrance. Clinical trials have reported the use of this compound in the treatment of lymphoedema and chronic venous disease (CVD). This compound reduces the accumulation of protein and oedema fluid in damaged tissues by stimulating phagocytosis and the production of proteolytic enzymes [29,30,31]. Coumarin exhibits sedative, anti-inflammatory, and spasmolytic effects. It may inhibit the proliferation of cancer cells, including malignant prostate cancer (DU145, LNCaP) and renal cancer (786-O, A-498) in humans [32]. In our previous research, we observed that coumarin exhibits antiplatelet activity by inhibiting ADP- and collagen-induced platelet aggregation [33]. However, despite its numerous health-promoting properties, its content in consumable products and the dosage must be controlled. Synthetic coumarin has been used as a flavouring agent in food, alcoholic beverages, and tobacco products. Its use was banned in the United States in 1954 by the Food and Drug Administration (FDA) due to concerns that it may exhibit hepatotoxic, genotoxic, and carcinogenic effects in humans, based on animal laboratory studies [34,35]. The toxic effects of coumarin on the human body have so far not been conclusively confirmed. Felter et al. [35], based on an analysis of multiple studies, concluded that hepatotoxicity effects were observed only in patients treated with very high doses of coumarin (in the order of grams per day). In contrast, no adverse effects were reported in individuals exposed to coumarin from natural dietary sources or cosmetics [35]. The European Food Safety Authority (EFSA) established a tolerable daily intake (TDI) of 0.1 mg/kg body weight based on the no observed adverse effect level (NOAEL) in animal studies. In 2008, the European Commission set the permissible dose of coumarin in food products at 2 mg/kg (with some exceptions). Currently, there are no legal regulations regarding herbs or spices [36]. It is worth noting that sweet clover, particularly white sweet clover, has diverse applications in agriculture and apiculture. This plant is valued for its use as animal fodder and green manure and its exceptional capacity for honey production [37].

Of the available studies, most focused primarily on the analysis of the antioxidant activity, phenolic compounds, or coumarin content, usually considering the whole plant without distinguishing between individual plant organs. Therefore, in the present study, the profile of phenolic compounds and antioxidant activity were analysed, taking into account the species (white and yellow sweet clover), morphological parts (flowers, leaves, and stems), and the drying process (fresh and dried plants). To the best of our knowledge, this has not been analysed previously. The results allowed us to compare *M. albus* to *M. officinalis*, which is known for its beneficial properties, and to identify an extract rich in bioactive compounds. Importantly, the analyses were preceded by the optimisation of the extraction process.

## 2. Results and Discussion

### 2.1. The Optimisation of the Extraction Process

The first stage of the research involved optimising the extraction process from the fresh and dried flowers of white and yellow sweet clover. This part of the study focused on the characteristic metabolite of this species, coumarin, as well as compounds regarded as its precursors: *o*-coumaric acid and melilotic acid [5]. Four different solvents were used: 50% (*v*/*v*) ethanol solution, 80% (*v*/*v*) methanol solution, boiling water, and a boiling 10% (*w*/*v*) NaCl solution. Three extraction methods were applied: grinding with a solvent in a mortar, shaking in a laboratory shaker, and ultrasound-assisted extraction (UAE). The selection of solvents and methods was based on the available literature [38,39,40,41]. The obtained results are presented in Table 1. The highest coumarin content was achieved by using boiling water and a boiling 10% (*w*/*v*) NaCl solution as solvents, combined with grinding the sample in a mortar. Boiling water extraction appeared particularly interesting from a nutritional perspective, as herbs are most commonly consumed as infusions. A high extraction efficiency was also achieved using 50% *v*/*v* ethanol, considering not only coumarin but also melilotic acid. As is well known, ethanol is used to produce plant extracts used in folk medicine and pharmacy. The extraction procedure itself had a significant impact on the results, whereas the choice of solvent was of secondary importance (*p* < 0.05).

The efficiency of extraction decreased in the following order: grinding in a mortar > ultrasound-assisted extraction > shaking in a laboratory shaker. The fresh flowers contained significantly higher levels of coumarin compared to the dried flowers, regardless of the extraction procedure applied. The scientific literature indicates that the coumarin content in fresh plants may initially be low but increases following mechanical damage. The plant accumulates *o*-coumaric acid glucoside in its vacuoles, which is enzymatically converted to coumarin as a result of tissue damage [18,38]. *O*-coumaric acid was identified at very similar levels in the dried flowers of white sweet clover (0.17–0.21 mg/g) and yellow sweet clover (0.42–0.50 mg/g), but it was not detected in the fresh plant. Bourgaud et al. [38] compared seven different extraction procedures for the quantification of coumarin and glucosyl-*o*-hydroxycinnamic acid in *M. officinalis*. They used different solvents, including water, ethanol, methanol, ethyl acetate, diethyl ether, and chloroform, as well as various procedures, such as stirring with solvents (at boiling or room temperature) and refluxing with solvents. They observed that extraction with polar solvents was the most effective, with water extraction resulting in the highest total concentration of coumarin, which is consistent with our results. Celeghini et al. [39] optimised the extraction of coumarin from *Mikania glomerata* Spreng. (‘guaco’) leaves using various extraction procedures: maceration with a 50% *v*/*v* ethanol solution, ultrasound-assisted extraction with water–ethanol solutions (using different ultrasound exposure times and ethanol concentrations), an infusion with boiling water, and supercritical fluid extraction (SFE). They found that the best results, considering the time/yield ratio, were obtained using 50% *v*/*v* ethanol solutions with the ultrasound-assisted extraction method. They obtained a significantly lower coumarin content of 393.8 µg/mL (which corresponds to 3.94 mg/g of dry leaf powder) using an infusion with boiling water, whereas ultrasound extraction and maceration provided 656.2 µg/mL (6.56 mg/g) and 696.4 µg/mL (6.96 mg/g), respectively. The use of various solvents (80% *v*/*v* methanol, 80% *v*/*v* ethanol, acetonitrile, and chloroform) for effective coumarin extraction in bakery products was also analysed in the study by Sproll et al. [36]. They found that 80% *v*/*v* methanol combined with magnetic stirring was the most effective (0.075 mg/g in the bakery product matrix), whereas the least effective was acetonitrile (0.045 mg/g). In the study by Martino et al. [40], the content of *o*-coumaric acid in the flowers of yellow sweet clover ranged from 1.35 mg/g to 0.43 mg/g, while the content of melilotic acid varied between 8.68 mg/g and 3.46 mg/g, depending on the solvent used and the extraction time in the ultrasound-assisted solvent extraction method. In their study, the best results were obtained by extracting the sample for 120 min using a 50% (*v*/*v*) ethanol solution, while the weakest results were observed with a 10 min extraction using pure methanol. Under these conditions, the coumarin content was 3.62 mg/g and 1.82 mg/g, respectively. The high efficiency of the ultrasound-assisted extraction technique using ethanol as the solvent was also proven in the study conducted by Savić et al. [42], who analysed the total phenolic compounds content of wheatgrass (*Triticum aestivum* L.).

Considering the obtained results and the analysis of the literature data, a combined method was applied in further analyses: the plant material was ground in a mortar with a solvent and then subjected to ultrasound treatment using 50% *v*/*v* ethanol as the solvent.

### 2.2. Phenolic Compound Profile

In the sweet clover extracts, characteristic compounds were identified and classified into three groups: coumarin derivatives (coumarin, umbelliferone), phenolic acids (melilotic acid, *o*- and *p*-coumaric acids, and *o*-coumaric acid glycoside), and flavonoids (quercetin, hyperoside, and luteolin, as well as quercetin and kaempferol glycosides). The content of these compounds in the extracts decreased in the following order: flavonoids > coumarins > phenolic acids. The highest content of coumarin was found in fresh sweet clover flowers (Table 2), ranging from 12.36 to 26.30 mg/g. The content in the dried flowers varied between 5.31 and 9.74 mg/g. Incorrect information can often be found in the general literature and online sources claiming that the coumarin content increases during drying. However, as demonstrated in the conducted experiment, a decrease in the coumarin content occurs during the drying process. The coumarin content did not differ significantly between the sweet clover species, measuring 21.72 mg/g in *M. albus* and 18.64 mg/g in *M. officinalis*. The obtained results align with the findings of Maggi et al. [41], who analysed differences in the coumarin content in the fresh, liquid nitrogen-homogenised, and dried leaves of *Melittis melissophyllum* L. Although drying promotes coumarin biosynthesis due to tissue damage in the plant material, its volatility leads to significant losses during the process [41]. The content of umbelliferone was not determined in fresh flowers (Table 2). Its average content in white sweet clover was 0.16 mg/g, while in yellow sweet clover, it was 0.12 mg/g. No differences were observed between species (*p* < 0.05). Among the identified phenolic acids, the compound present in the highest concentration was *o*-coumaric acid glycoside. Additionally, no statistically significant differences (*p* < 0.05) were observed between species or in the effect of drying on the sample (average content of this compound: 4.46 mg/g). In dried flowers, higher levels of *o*-and *p*-coumaric acids were observed compared to fresh flowers, which may indicate transformations occurring during the drying process. The content of these compounds was identified as being at a low level. In dry samples, *o*-coumaric acid averaged 0.34 mg/g in *M. albus* and 1.00 mg/g in *M. officinalis*, while in fresh plants, it was below the limit of quantification (LOQ). The *p*-coumaric acid content in dry flowers averaged 0.16 mg/g in *M. albus* and 0.12 mg/g in *M. officinalis*, while in fresh flowers it was 0.13 mg/g and 0.07 mg/g, respectively. A slight reduction in the melilotic acid content was observed in fresh compared to dry flowers. However, as with the other compounds, no statistically significant differences were found between the analysed sweet clover species.

Sweet clover flowers, especially *M. albus*, are a rich source of flavonoids (Table 3). Their content decreased in the following order: hyperoside > quercetin glycoside > quercetin > luteolin > kaempferol glycoside. The content of flavonoids, except kaempferol glycoside, did not change significantly during the drying process, indicating the thermostability of these compounds. An important compound in this group is hyperoside, one of the quercetin glycosides. White sweet clover flowers contained over six times more of this compound compared to yellow sweet clover flowers. Moreover, *M. albus* did not contain kaempferol glycoside, which was detected in *M. officinalis* flowers. The other flavonoids were present at similar levels regardless of the species of sweet clover studied (*p* < 0.05). The identification of quercetin and kaempferol glycosides was based on the literature data and the UV-Vis spectra characteristic of the derivatives of these two compounds. Both quercetin and kaempferol are classified as flavonols. Quercetin exhibits characteristic bands at 256 and 372 nm, while kaempferol shows characteristic bands at 264 and 370 nm. Derivatives of flavonols, such as hyperoside, were identified based on the retention time (in comparison with analytical standards) and characteristic absorption maxima at 254 and 354 nm. Two distinct bands, one near λ = 250 nm and the other around 350 nm, are typically characteristic of flavones and/or flavonol-3 derivatives. Generally, if the first band is more intense than the second, it suggests the presence of a flavonol derivative, while if the second band is more intense, it indicates the presence of a flavone. Given that 3-*O*-glycosylated flavonols are characterised by specific UV-Vis spectra, the compounds analysed in this study were considered to be derivatives of quercetin and kaempferol based on their spectral profiles [43,44]. With the exception of flavonols, the only flavone identified was luteolin, with an average content in dried *M. albus* of 0.89 mg/g and in dried *M. officinalis* of 0.90 mg/g, whereas in fresh samples, the content was 0.56 mg/g and 0.70 mg/g, respectively.

The contents of coumarin, umbelliferone, and phenolic acids determined in the leaves of sweet clover are presented in Table 4. Similarly to the flowers, a lower content of coumarin was observed in dried leaves compared to flowers (approximately a 5-fold decrease). In white sweet clover, the coumarin content ranged between 0.80 mg/g and 3.24 mg/g, while in yellow sweet clover, it was at a similar level, ranging from 1.34 mg/g to 1.95 mg/g (in dried samples). It was not demonstrated that *M. officinalis* is a better source of this compound. Umbelliferone was identified in both fresh and dried leaves, unlike in the flowers. However, its content was at a low level, varying between 0.23 and 0.61 mg/g in dried leaves and 0.26 to 0.51 mg/g in fresh ones. No statistically significant differences were found between the species (*p* < 0.05). For the identified phenolic acids, no significant differences between the species were observed (*p* < 0.05). The phenolic acid identified in the highest quantity was melilotic acid, with a higher content noted in comparison to that of flowers. Its average content in *M. albus* was 5.70 mg/g in dried leaves and 11.66 mg/g in fresh leaves, while in *M. officinalis*, it was 5.50 mg/g and 8.13 mg/g, respectively. *O*-coumaric acid and *p*-coumaric acid were identified at low levels and only in dried samples. The content of *o*-coumaric acid glycoside was found to be lower than that in flowers, ranging from 0.61 to 2.01 mg/g (dried leaves) and 0.49 to 2.96 mg/g (fresh leaves).

The flavonoid profile found in the extracts of sweet clover leaves was similar to that described for the flowers (Table 5). However, the content of these compounds in the leaves was several times lower than in the flowers (approximately fourfold), with the highest flavonoid content observed in the leaves of *M. albus*. Similarly, no significant changes in the content of these compounds were observed during the drying process. Interestingly, unlike in flowers, no higher content of hyperoside was observed in *M. albus* compared to *M. officinalis*. Its average content was 4.27 mg/g in *M. albus* and 3.33 mg/g in *M. officinalis* in dried samples and 3.89 mg/g and 2.47 mg/g in fresh samples. Other flavonoids were detected at significantly lower levels. The species-specific presence of kaempferol glycoside in the leaves of yellow sweet clover was confirmed.

The results of the identification of phenolic compounds in sweet clover stems are presented in the Supplementary Materials: coumarins and phenolic acids (Table S1) and flavonoids (Table S2). The overall compound profile was similar to that of the other organs. However, the identified compounds were generally present at low levels. The average coumarin content in the dried stems of *M. albus* was 0.76 mg/g, while in *M. officinalis*, it was 0.66 mg/g, which shows that the content was approximately ten times lower than that in flowers and about two times lower than that in leaves. Similarly, higher levels of this compound were observed in fresh material. The content of umbelliferone was, on average, 0.10 mg/g in both dried and fresh stems. No differences were observed between species (*p* < 0.05). Phenolic compounds were identified at levels below 1 mg/g, with the exception of melilotic acid, whose content in dried white sweet clover ranged from 0.43 to 1.30 mg/g and in yellow sweet clover from 0.30 to 2.79 mg/g. Similarly to coumarin compounds, no significant differences were observed between *M. albus* and *M. officinalis*. The flavonoid content in sweet clover stems was the lowest, while maintaining a compound profile analogous to that of the flowers (Table S2). No alterations during the drying process were detected. With the exception of hyperoside in the stems of *M. albus*, the remaining flavonoids were identified at levels lower than 1 mg/g. Furthermore, the presence of a species-specific kaempferol glycoside in the stems of yellow sweet clover was verified.

Considering the total identified phenolic compounds, flowers proved to be the richest source. Among the analysed samples, *M. albus* exhibited a higher phenolic content than *M. officinalis*. In dried flowers, the content was 35.20 mg/g in *M. albus* and 24.91 mg/g in *M. officinalis*, while fresh flowers contained 47.24 mg/g and 30.92 mg/g, respectively. Leaves also contained significant levels of phenolic compounds, though these were lower than those in flowers, with 14.08 mg/g and 12.74 mg/g in dried samples and 26.77 mg/g and 20.94 mg/g in fresh samples for white and yellow sweet clover, respectively. The stems had the lowest phenolic content, measuring 3.52 mg/g and 3.49 mg/g in dried samples and 6.95 mg/g and 5.07 mg/g in fresh samples for *M. albus* and *M. officinalis*, respectively.

Studies conducted in recent years have demonstrated that plants of the *Melilotus* genus exhibit considerable variability in the coumarin content, depending on the species and the country of origin. Kitchen et al. [45] analysed methanolic extracts obtained from the leaves of 13 Melilotus species, including *M. officinalis*, *M. albus*, *M. dentatus*, and *M. elegans*, collected at the flowering stage. The coumarin content ranged from 0.05% to 1.04% of dry matter, with significant variation observed within individual species. In the study by Nair et al. [46], the coumarin content was determined in the fresh leaves of 15 *Melilotus* species collected during the flowering stage, with the results for *M. albus* ranging from 0.17% to 1.3% of dry matter and for *M. officinalis* ranging from 0.16% to 0.61% of dry matter. The compound was extracted using ultrasound-assisted extraction with water as the solvent. Abbasi et al. [47] reported the coumarin content in the leaves of two-year-old white and yellow sweet clover, ranging from 0.09% to 5.25% (samples were analysed from over 190 locations using a fluorometric method). Zhang et al. [10] analysed the variability of coumarin in *M. officinalis* and *M. albus* collected from 93 different locations. Ultrasound-assisted extraction was used to obtain 50% ethanol extracts, and the coumarin content was determined using a UV-Vis spectrophotometric method. In *M. officinalis*, the content ranged from 0.3% to 1.5%, while in *M. albus*, it ranged from 0.2% to 1.3%, with significant variation observed within each species. In the general literature, it has been reported that *Melilotus albus* contains less coumarin compared to *M. officinalis*, which is considered a medicinal plant largely due to its coumarin content. In the cited publications, as well as in our own studies, no significant differences were found in the coumarin content between these two species. Comparing our results with those of other authors is challenging, as their data are expressed as percentages and different extraction and analytical techniques were used.

The obtained results of the phenolic compound profile analysis are consistent with reports from other authors. As noted by Witowska-Banaszczak et al. [18], both *M. albus* and *M. officinalis* contain coumarin, umbelliferone, melilotic acid, and *p*-coumaric and *o*-coumaric acids, as well as derivatives of quercetin and kaempferol (quercetin 3-rhamnosyl-(1-6)-galactoside 7-*O*-rhamnoside and kaempferol 3-galactosyl-(1-6)-glucoside 7-*O*-rhamnoside). Bubenchikova and Drozdova [48] conducted an analysis of phenolic compounds in the leaves of *M. officinalis*. The compound profile they obtained differed from that observed in the present study. However, the extraction was performed using 70% ethanol by boiling with reflux in a water bath. The authors identified 18 compounds, with ferulic acid being the most abundant (25.57% of the total content), followed by arbutin and caffeic acid (14.91% and 12.57%, respectively). Moreover, the presence of hyperoside (5.86%), luteolin (0.03%), quercetin (0.02%), and coumarin (3.85%) was confirmed. Liu et al. [27] analysed the phenolic compounds in the aerial parts of *M. officinalis* using 70% ethanol as the extraction solvent and isolated coumarin, salicylic acid, betaine, fumaric acid, caffeic acid, luteolin, quercetin, and glycosides of *p*-hydroxybenzoic acid. They isolated compounds using a column chromatographic (CC) method, and then fractions were purified using semipreparative HPLC. Similarly to the findings of Bubenchikova and Drozdova [48], the exact content of the identified compounds was not determined. Safarpour et al. [15] demonstrated that an aqueous extract of *M. officinalis* contained coumarin (289.0 mg/L), *p*-coumaric acid (126.0 mg/L), cinnamic acid (548.9 mg/L), catechin (99.1 mg/L), and quercetin (1.1 mg/L). Differences between the present study and the results obtained by other authors are likely due to variations in the extraction methods for phenolic compounds. As can be observed, there is a lack of studies and publications focusing on the analysis of the phenolic compound profile of *M. albus*.

### 2.3. Antioxidant Activity

The antioxidant activity (assessed using the DPPH**·** and FRAP methods) and the total phenolic content (TPC method) were determined for dried flowers and leaves (ground in a mortar with 50% *v*/*v* ethanol and treated with ultrasound). These aerial parts of the plant were chosen due to their significantly higher bioactive compound content compared to the stems. Dried material was selected because herbs in this form can be stored for a longer period, are more stable, and are less prone to fermentation and decay. The results are presented in Table 6. Higher antioxidant activity was observed in leaf extracts compared to flower extracts in both the FRAP and DPPH**·** assays. Particularly significant differences were noted when using the DPPH**·** radical scavenging method, where the antioxidant activity was, on average, twice as high. The average antioxidant activity was as follows: for the *M. albus* flowers, 53.77 µmol TE/g (FRAP test) and 49.18 µmol TE/g (DPPH**·** test); and for the *M. officinalis* flowers, 82.72 µmol TE/g and 73.26 µmol TE/g, respectively. On the other hand, for the *M. albus* leaves, the average values were 71.97 µmol TE/g and 114.96 µmol TE/g, and for the *M. officinalis* leaves, they were 91.49 µmol TE/g and 177.66 µmol TE/g in the FRAP and DPPH**·** tests, respectively. Simultaneously, the level of phenolic compounds in the leaf extracts was approximately 30% higher compared to that in the flower extracts (on average, 22.10 mg GAE/g and 29.07 mg GAE/g in *M. albus* and 32.56 mg GAE/g and 42.19 mg GAE/g in *M. officinalis* for the flowers and leaves, respectively). The content of phenolic compounds was highly correlated with the antioxidant activity (r = 0.85 and 0.87, respectively, in the DPPH**·** and FRAP assays). The correlation between the antioxidant activity measured by the DPPH**·** and FRAP assays was r = 0.81. Among the analysed phenolic compounds, the contents of melilotic acid and umbelliferone were positively correlated with the DPPH**·** assay (r = 0.53 and 0.55, respectively). These results indicate that the leaf extracts of sweet clover, unlike the flowers, contain not only phenolic compounds but also other bioactive substances exhibiting antioxidant activity. Considering that the DPPH**·** radical dissolves only in organic solvents (particularly in alcoholic solutions), the assay is more sensitive to hydrophobic antioxidants [49].

The obtained results may suggest the involvement of other biologically active substances with hydrophobic properties in shaping the antioxidant activity of leaf extracts. The high antioxidant activity of plant extracts (including algae such as *Arthrospira platensis*, *Chlorella vulgaris*, *Tetraselmis suecica*, and *Phaeodactylum tricornutum*) rich in chlorophyll pigments, measured using the DPPH**·** assay, has been observed in studies by other authors [50]. Similarly, Alvarez-Parrilla et al. [51] demonstrated that reducing the chlorophyll content in various varieties of jalapeño and serrano chilli peppers significantly decreased their antioxidant activity against the DPPH**·** radical.

Analysing interspecies differences, it was observed that both the flowers and leaves of *Melilotus officinalis* exhibited higher antioxidant activity and a higher total phenolic content compared to white sweet *Melilotus albus* (*p* < 0.05). The reducing power measured using the FRAP assay was 62% and 27% higher for the flowers and leaves of *M. officinalis*, respectively, while the phenolic compound content was 43% and 59% higher, respectively.

Safarpour et al. [15] determined the antioxidant activity of aqueous extracts of *M. officinalis* to be at the level of 2.91 µmol/g, which is significantly lower than that in the present study. In the study by Mladenović et al. [25], the antioxidant activity of aqueous, acetone, ether, and ethanol extracts of yellow sweet clover against the DPPH**·** radical was analysed. The results were expressed as the % inhibition and, therefore, cannot be directly compared with the present study. However, they demonstrated variability in the activity depending on the solvent used and the extract concentration. The most effective in neutralising the DPPH**·** radical was the aqueous extract at a concentration of 1000 µg/mL, while the weakest was the acetone extract at a concentration of 62.5 µg/mL. They also determined the total phenolic content, which ranged from 36.25 mg GAE/g extract (aqueous extract) to 16.37 mg GAE/g (ether extract). Borhani et al. [28] determined the antioxidant activity and phenolic compound content in aqueous and ethanol extracts of the leaves, stems, and roots of *Melilotus officinalis* collected from various locations in two northern provinces of Iran. They observed that the flowers had the highest phenolic compound content, averaging 31.2 mg GAE/g (ethanol extracts) and 19.64 mg GAE/g (aqueous extracts). Furthermore, the antioxidant activity determined by them against the DPPH**·** radical was 13.1 IC50 mg/mL on average. Likewise, the antioxidant activity of *M. albus* is poorly studied. Stefanović et al. [24] investigated the antioxidant activity using the DPPH**·** and FRAP methods, as well as the total phenolic content, in ethanol, acetone, and ethyl acetate extracts from the aerial parts of *M. albus*. As in the study by Mladenović et al. [25], it was shown that both the antioxidant activity and reducing power depend on the extract concentration and the solvent used. Due to the differences in the methodology and the way the results were presented, a comparison with the present study is not possible. However, the total phenolic content determined by Stefanović et al. [24] ranged from 14.80 mg GAE/g (ethanol extract) to 28.80 mg GAE/g (acetone extract). The results cited are in agreement with the findings of the present study.

### 2.4. Multivariate Statistical Analysis

To determine the relationships between the studied species of sweet clover and the selected aerial parts of the plant (flowers, leaves), principal component analysis (PCA) and cluster analysis (CA) combined with a heatmap were conducted. This analysis was based on the obtained results of the antioxidant activity and the content of individual phenolic compounds.

#### 2.4.1. Principal Component Analysis (PCA)

**Figure 1:**
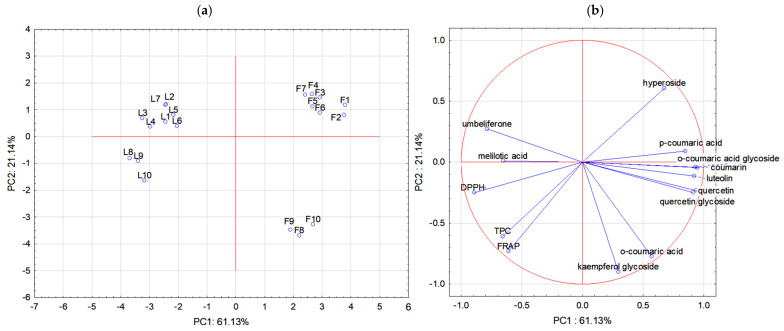
Principal component analysis results: (**a**) projection of analysed flowers and leaves of *Melilotus albus* and *Melilotus officinalis* (F1–F7: *M. albus* flowers; F8–F10: *M. officinalis* flowers; L1–L7: *M. albus* leaves; L8–L10: *M. officinalis* leaves); (**b**) selected variables (individual phenolic compounds, antioxidant activity, and total phenolic compound content—TPC) as function of PC1 vs. PC2.

Based on the Kaiser criterion, 14 variables were reduced to two principal components which explained 82.27% of the variance (PC1: 61.13%; PC2: 21.14%). The relationships obtained between the analysed parameters and the flowers and leaves of sweet clover are shown in Figure 1.

The analysed samples were clearly divided into four groups. The first principal component was associated with the content of most phenolic compounds, such as coumarin, *o*-coumaric acid glycoside, *p*-coumaric acid, hyperoside, quercetin, luteolin, quercetin glycoside, and weakly with *o*-coumaric acid (positively), and melilotic acid, hyperoside, the TPC, DPPH**·**, and the FRAP (negatively). The second principal component was associated with *o*-coumaric acid and kaempferol glycoside and, similarly to the first, with the TPC, DPPH**·**, and the FRAP (negatively).

The flowers of *M. albus* are located in the top-right corner of the plot in a very tight cluster, indicating their close similarity. The characteristic compound for the flowers of white sweet clover was hyperoside. The flowers of *M. officinalis* are positioned at the bottom of the plot, also on the right side. The flowers of yellow sweet clover exhibited the highest content of *o*-coumaric acid and kaempferol glycoside. The leaves of sweet clover are located on the left side of the plot. As previous studies have shown, they exhibited higher antioxidant activity (especially yellow sweet clover) and a higher content of umbelliferone. It can also be observed that the similarity between the leaves of yellow and white sweet clover was greater than that between the flowers.

#### 2.4.2. Hierarchical Clustering Analysis (HCA) and Heatmap Visualisation

The analysis was performed using the Euclidean distance as the measure of dissimilarity and Ward’s method for clustering. The importance of the variables was determined based on the C&RT model. Most phenolic compounds (coumarin, umbelliferone, quercetin, *o*-coumaric acid glycoside, *p*-coumaric acid, quercetin glycoside, luteolin) and the antioxidant activity measured using the DPPH**·** assay were identified as equally important (importance score: 1). Slightly lower values were observed for hyperoside and the reducing power determined using the FRAP method (importance score: 0.95).

**Figure 2:**
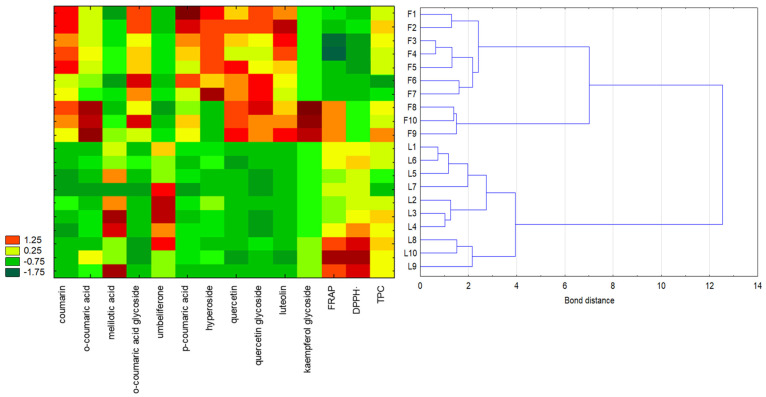
Hierarchical clustering analysis and heatmap visualisation of the analysed flowers and leaves of *Melilotus albus* and *Melilotus officinalis* (F1–F7: *M. albus* flowers; F8–F10: *M. officinalis* flowers; L1–L7: *M. albus* leaves; L8–L10: *M. officinalis* leaves), based on the content of individual phenolic compounds, the antioxidant activity, and the total phenolic content (TPC). The cluster analysis was performed using standardised data. The colours on the heatmap represent the values of individual parameters, with red indicating high values and dark green indicating low values.

The results of the cluster analysis confirmed the relationships observed using PCA. Flowers and leaves were divided into two distinct clusters, with further subclusters representing the flowers and leaves of *Melilotus officinalis* and *Melilotus albus* (Figure 2). Samples with the most similar values of the determined parameters were positioned closest to each other. The heatmap colours made it possible to perform a comparative analysis of the specific parameter levels, with red indicating the highest value of the studied parameter and green representing the lowest. This data visualisation clearly highlights which sweet clover samples exhibited the highest or lowest content of a given compound. For example, the highest content of coumarin was found in samples F1, F2, and F5 (flowers of *M. albus*, sample numbers 1, 2, and 5), while the highest content of *o*-coumaric acid was observed in F8, F9, and F10 (flowers of *M. officinalis*, sample numbers 8, 9, and 10). Multivariate statistics could therefore be effectively employed to differentiate plant species based on the content of biologically active compounds.

## 3. Materials and Methods

### 3.1. Chemicals and Reagents

The determinations were performed using analytical grade reagents intended for liquid chromatography: methanol (J.T. Baker, Phillipsburg, NJ, USA), acetonitryl (CHROMASOLV^®^ gradient grade, ≥99.9%, from Sigma-Aldrich, St. Louis, MO, USA), and ammonium acetate and acetic acid (Sigma-Aldrich, St. Louis, MO, USA). Deionised water from the deioniser model HLP 5P was used (Hydrolab, Poznan, Poland). Phenolic acid analytical standards of HPLC grade for coumarin, *o*-coumaric acid, *p*-coumaric acid, melilotic acid, quercetin, kaempferol, hyperoside, umbelliferone, luteolin, and gallic acid were purchased from Sigma-Aldrich (St. Louis, MO, USA). Other reagents used for the analyses were of analytical-grade purity: 2,2-diphenyl-1-picrylhydrazyl (DPPH·), Folin–Ciocalteu reagent, 2,4,6-tri(2-pyridyl)-s-triazine (TPTZ), 6-hydroxy-2,5,7,8-tetramethylchroman-2-carboxylic acid (Trolox), sodium carbonate, aluminium chloride, and sodium acetate, obtained from Sigma-Aldrich (St. Louis, MO, USA), and ethanol and sodium chloride from POCH (Gliwice, Polska).

### 3.2. Plant Material

**Figure 3:**
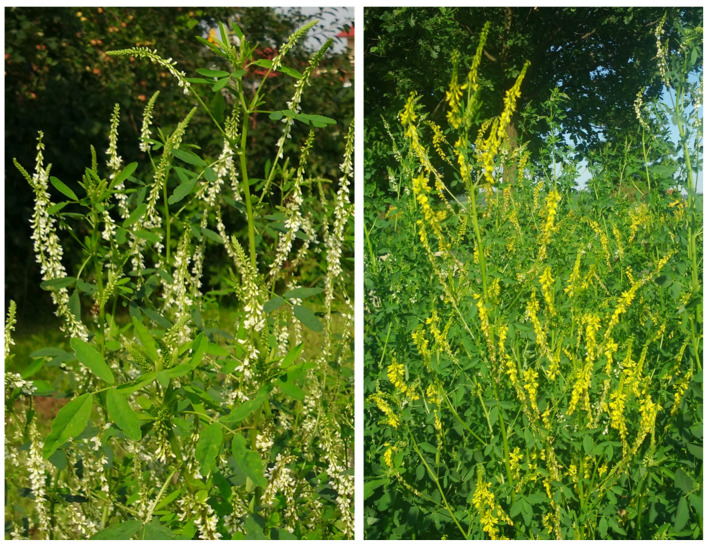
White sweet clover (*Melilotus albus*) and yellow sweet clover (*Melilotus officinalis*) in the left and right photos, respectively (source: author’s own archive).

The plants were collected at the flowering stage in July in 2021 and 2022 from 10 organic agricultural crops located in the Podkarpackie Voivodeship, situated in the southeastern part of Poland. Table 7 provides detailed information regarding the analysed species, including the exact location of the crops and the year in which the studied material was collected. No fertilisers and chemicals were used during the study. Additionally, the farmers sowed certified seed material. Forty plants were collected each time. The botanical identification of each plant was confirmed using the Department of Crop Production archive at https://atlas.roslin.pl/listing/all/1/t_genus/Melilotus (accessed on 10 January 2025). A voucher specimen (Table 7) of the plant was deposited in the Department archive. Photographs of the analysed species, *M. albus* and *M. officinalis*, are presented in Figure 3 (showing an example of the plants collected from locations 5 and 9).

The collected plant material was divided into flowers, leaves, and stems. Part of the material was immediately used for analysis, while the remainder was dried to a moisture content of approximately 4% at room temperature (average of 20 ± 2 °C) in a well-ventilated room. The dried material was sealed in airtight plastic containers and stored without exposure to light until analysis. The moisture content of both the fresh and dried material was determined using a moisture analyser (Radwag MA 50.R, Puszczykowo, Poland). Immediately before analysis, the plant material was ground using a laboratory mill (A11 IKA, Königswinter, Germany).

### 3.3. Extract Preparation

Various extraction conditions were tested, with 2 g of the tested plant material extracted with 20 mL of the selected solvent using different methods:

- Solvent type: 50% (*v*/*v*) ethanol solution, 80% (*v*/*v*) methanol solution, boiling water, and 10% NaCl (*w*/*v*) boiling solution.
- Extraction methods: grinding in a mortar (without shaking), shaking in a rotary shaker (Biosan ES-20/60, Riga, Latvia) for 30 min at 200 rpm, and ultrasound-assisted extraction using an ultrasonic bath (U-504 Ultron, Moorpark, CA, USA) at 560 W and 40 kHz for 30 min at room temperature (20 ± 2 °C).

For the primary analysis, the optimal extraction method was selected, using an ethanol–water solvent (1:1 ratio) with the plant sample ground in a mortar, followed by ultrasound-assisted extraction. As in the preliminary tests, 2 g of the material was extracted with 20 mL of the solvent. The samples were then centrifuged at 3500 rpm for 10 min (MPW-260, Warsaw, Poland), and the supernatant was filtered through 0.22 µm nylon filters (Merck Millipore, Darmstadt, Germany). Each extraction procedure was repeated twice.

### 3.4. HPLC Analysis

The identification of phenolic compounds was performed using a SYKAM S600 high-performance liquid chromatograph with a photodiode array detector (PDA) (Ersing, Germany). Analysis conditions: we used a Bionacom Velocity STR C18 column (3.0 × 100 mm; 2.5 μm), thermostated at 40 °C, with an injection volume of 20 μL, a flow rate of 0.5 mL/min, and detection at λ = 280 nm (e.g., coumarin, hydroxybenzoic acids), 320 nm (e.g., hydroxycinnamic acids), and 360 nm (mainly flavonoids). The wavelength was selected based on the maximum absorption of the compound, according to the literature data. Mobile phase composition: we used an aqueous solution of 5 mM ammonium acetate and 0.2% (*v*/*v*) acetic acid (phase A) and acetonitrile/methanol (1:2 *v*/*v*) (phase B). A gradient elution was applied during the analysis: 70% A (2 min), 35% A (13 min), and 70% A again (5 min). Compounds were identified by comparing the UV spectra and retention times of the separated substances with the corresponding standards. Quantitative analysis was performed based on the calibration curves of individual standards. For each standard, five-point calibration curves were prepared in the range of 0.005 to 0.1 mg/mL (r^2^ ≥ 0.9989). The content of *o*-coumaric acid glycoside was expressed as *o*-coumaric acid, while quercetin and kaempferol were expressed as quercetin equivalents, respectively. The obtained results were expressed as milligrams per gram (mg/g) of the plant’s dry weight (dw). An example of the obtained chromatogram is presented in the Supplementary Materials (Figure S1: HPLC-PDA chromatogram of phenolic compounds of dried *Melilotus albus* flower extracts recorded at 280 nm).

The developed quantitative methods were validated in terms of the linearity range, limit of detection (LOD), limit of quantitation (LOQ), and precision, expressed as the percentage relative standard deviation (%RSD) of the retention times and peak areas, according to the International Council for Harmonisation (ICH) Guidance for Industry [52]. The LOD and LOQ were determined based on the signal-to-noise (S/N) ratio, with S/N = 3 for the LOD and S/N = 10 for the LOQ. The reproducibility of the method was assessed using three selected concentrations of each standard, as well as the real sample (*M. albus* flower extract), with three repetitions performed within a single day for each concentration. The procedure was repeated for five consecutive days. The precision was expressed as the percentage relative standard deviation (%RSD) for both the intra-day and inter-day repeatability. The validation results are presented in Table S3.

### 3.5. Analysis of Antioxidant Activity

The antioxidant activity was determined using the FRAP method (Ferric Ion-Reducing Antioxidant Power) and the DPPH· (2,2-diphenyl-1-picrylhydrazyl) radical scavenging activity, following the procedure described by Sowa et al. [53]. The antioxidant activity results were expressed as Trolox equivalents (TEs) per gram of the dry weight of the plant material (µmol TE/g dw), based on the calibration curves of the Trolox solution in the concentration ranges of 15–200 nmol (FRAP) and 5–60 nmol (DPPH·). The calibration curve for the FRAP was y = 0.0248x − 0.034 (r^2^ = 0.998), and for DPPH, it was y = 1.5232x − 4.7015 (r^2^ = 0.988). The analyses were performed using a UV-Vis spectrophotometer (Biomate 3, Thermo, Madison, WI, USA). The reagents were purchased from Sigma Aldrich (St. Louis, MO, USA).

### 3.6. Total Phenolic Content (TPC)

The total phenolic content (TPC) was measured using the Folin–Ciocalteu reagent, according to the procedure outlined by Sowa et al. [53]. A calibration curve (y = 0.0543x + 0.0195, r^2^ = 0.998) was prepared for gallic acid in the concentration range of 0–200 mg/mL. The content of phenolic compounds was expressed as gallic acid equivalents (GAEs) per gram of the dry weight of the plant material (mg GAE/g dw). The analyses were conducted using a UV-Vis spectrophotometer (Biomate 3, Thermo, Madison, WI, USA), with reagents sourced from Sigma Aldrich (St. Louis, MO, USA).

### 3.7. Statistical Analysis

All analyses were performed in triplicate. The results were presented as the mean ± the standard deviation (SD). Statistically significant differences between the applied extraction methods were determined using a two-way ANOVA (*p* < 0.05), followed by Tukey’s test. Differences between the sweet clover species based on the analysed parameters were assessed using a one-way ANOVA (*p* < 0.05) and Tukey’s test (for a different N). To illustrate the relationships between the analysed species and their morphological parts, principal component analysis (PCA) and hierarchical clustering analysis (HCA) with a heatmap visualisation were performed. Statistical analyses were conducted using Statistica 13.1 software (StatSoft, Inc., Tulsa, OK, USA).

## 4. Conclusions

It has been demonstrated that *Melilotus albus*, similarly to *Melilotus officinalis*—a valuable medicinal herb—is a rich source of phenolic compounds and exhibits strong antioxidant activity. The qualitative profile of the analysed compounds was similar for both species, with differences observed in the content of individual fractions. The most abundant source of these compounds was the flowers, followed by the leaves and, lastly, the stems. However, it is important to note that no statistically significant differences were observed between the two species. During this study, special attention was given to coumarin, the most characteristic metabolite of plants belonging to the *Melilotus* species, which is responsible for a number of health-promoting properties, but the content of which must be carefully monitored. The highest concentration of coumarin was found in the flowers. Additionally, it was shown that *M. officinalis* is not a richer source of this compound, as commonly believed. A significant decrease in the coumarin levels was observed in dried plant organs compared to fresh ones. The flowers of white sweet clover exhibited higher levels of hyperoside than those of yellow sweet clover. In contrast, yellow sweet clover contained a kaempferol glycoside, which was absent in the flowers of white sweet clover. It was also observed that the leaves of *Melilotus* species contained higher levels of melilotic acid and umbelliferone and exhibited greater antioxidant activity compared to the flowers.

The profile characteristics and assessment of the content of bioactive compounds in the flowers and leaves of white sweet clover, due to the greater prevalence of the cultivation of this species, provide a good basis for continuing research on testing the health-promoting properties of *M. albus* extracts using various biological models.

## Figures and Tables

**Table 1:** Effect of extraction conditions on content of coumarin and its precursors in extracts of dried and fresh white and yellow sweet clover flowers, determined using HPLC-PDA method.

Methods	Solvent	Coumarin [mg/g]	*o*-Coumaric Acid [mg/g]	Melilotic Acid [mg/g]
**Dried *Melilotus albus* flowers**				
Grinding in a mortar with a solvent	50% (*v*/*v*) ethanol	10.36 ± 0.11 ^a^	0.21 ± 0.01 ^a^	2.26 ± 0.11 ^a^
	80% (*v*/*v*) methanol	2.42 ± 0.19 ^b^	0.19 ± 0.02 ^a^	2.26 ± 0.20 ^a^
	boiling water	12.00 ± 0.90 ^c^	0.19 ± 0.01 ^a^	1.88 ± 0.05 ^a,c^
	boiling 10% (*w*/*v*) NaCl	11.32 ± 0.07 ^a^	0.17 ± 0.08 ^a^	0.99 ± 0.06 ^b^
Shaking in a laboratory shaker	50% (*v*/*v*) ethanol	3.25 ± 0.34 ^b^	0.18 ± 0.02 ^a^	2.28 ± 0.02 ^a^
	80% (*v*/*v*) methanol	2.16 ± 0.07 ^b^	0.18 ± 0.03 ^a^	2.05 ± 0.02 ^a^
	boiling water	2.80 ± 0.39 ^b^	0.17 ± 0.02 ^a^	1.90 ± 0.32 ^a^
	boiling 10% (*w*/*v*) NaCl	2.33 ± 0.11 ^b^	0.19 ± 0.02 ^a^	1.44 ± 0.12 ^b,c^
Ultrasound-assisted extraction	50% (*v*/*v*) ethanol	5.87 ± 0.17 ^d^	0.20 ± 0.02 ^a^	2.29 ± 0.14 ^a^
	80% (*v*/*v*) methanol	2.21 ± 0.04 ^b^	0.19 ± 0.01 ^a^	2.15 ± 0.05 ^a^
	boiling water	3.82 ± 0.12 ^b^	0.18 ± 0.05 ^a^	1.83 ± 0.06 ^a,c^
	boiling 10% (*w*/*v*) NaCl	5.64 ± 0.04 ^d^	0.20 ± 0.01 ^a^	0.98 ± 0.12 ^b^
**Dried *Melilotus officinalis* flowers**				
Grinding in a mortar with a solvent	50% (*v*/*v*) ethanol	7.94 ± 0.14 ^a^	0.46 ± 0.01 ^a^	2.67 ± 0.03 ^a,c^
	80% (*v*/*v*) methanol	2.18 ± 0.25 ^b^	0.48 ± 0.05 ^a^	1.29 ± 0.12 ^a,b^
	boiling water	9.56 ± 0.24 ^c^	0.50 ± 0.04 ^a^	1.47 ± 0.08 ^a^
	boiling 10% (*w*/*v*) NaCl	9.27 ± 0.18 ^c^	0.45 ± 0.04 ^a^	1.59 ± 0.18 ^a,b^
Shaking in a laboratory shaker	50% (*v*/*v*) ethanol	2.89 ± 0.05 ^b,f^	0.44 ± 0.02 ^a^	1.86 ± 0.14 ^a^
	80% (*v*/*v*) methanol	1.99 ± 0.04 ^b^	0.42 ± 0.03 ^a^	1.28 ± 0.65 ^a^
	boiling water	3.10 ± 0.02 ^d,f^	0.46 ± 0.03 ^a^	1.35 ± 0.05 ^a^
	boiling 10% (*w*/*v*) NaCl	2.22 ± 0.01 ^b^	0.48 ± 0.01 ^a^	1.34 ± 0.12 ^a^
Ultrasound-assisted extraction	50% (*v*/*v*) ethanol	5.54 ± 0.06 ^e^	0.45 ± 0.01 ^a^	1.36 ± 0.01 ^a^
	80% (*v*/*v*) methanol	2.10 ± 0.02 ^b^	0.47 ± 0.01 ^a^	1.30 ± 0.02 ^a,b^
	boiling water	5.30 ± 0.11 ^e^	0.46 ± 0.02 ^a^	1.20 ± 0.35 ^a,b^
	boiling 10% (*w*/*v*) NaCl	5.82 ± 0.05 ^e^	0.49 ± 0.03 ^a^	1.25 ± 0.24 ^a,b^
**Fresh *Melilotus albus* flowers**				
Grinding in a mortar with a solvent	50% (*v*/*v*) ethanol	18.77 ± 0.11 ^a^	<LOQ	0.98 ± 0.03 ^a,b^
	80% (*v*/*v*) methanol	19.76 ± 0.22 ^a^	<LOQ	1.24 ± 0.03 ^a^
	boiling water	22.32 ± 0.52 ^b^	<LOQ	1.10 ± 0.02 ^a^
	boiling 10% (*w*/*v*) NaCl	23.66 ± 0.11 ^c^	<LOQ	1.27 ± 0.11 ^a^
Shaking in a laboratory shaker	50% (*v*/*v*) ethanol	18.67 ± 0.08 ^a^	<LOQ	1.02 ± 0.04 ^a^
	80% (*v*/*v*) methanol	19.76 ± 0.12 ^a^	<LOQ	1.43 ± 0.05 ^a,c^
	boiling water	21.40 ± 0.10 ^b^	<LOQ	1.03 ± 0.04 ^a^
	boiling 10% (*w*/*v*) NaCl	22.12 ± 0.22 ^b^	<LOQ	1.16 ± 0.04 ^a^
Ultrasound-assisted extraction	50% (*v*/*v*) ethanol	18.71 ± 0.11 ^a^	<LOQ	0.87 ± 0.11 ^a,b^
	80% (*v*/*v*) methanol	19.34 ± 0.13 ^a^	<LOQ	1.21 ± 0.14 ^a^
	boiling water	21.72 ± 0.09 ^b^	<LOQ	1.18 ± 0.11 ^a^
	boiling 10% (*w*/*v*) NaCl	22.35 ± 0.22 ^b^	<LOQ	1.16 ± 0.02 ^a^
**Fresh *Melilotus officinalis* flowers**				
Grinding in a mortar with a solvent	50% (*v*/*v*) ethanol	17.67 ± 0.12 ^a^	<LOQ	1.46 ± 0.13 ^a^
	80% (*v*/*v*) methanol	17.62 ± 0.16 ^a^	<LOQ	1.40 ± 0.13 ^a^
	boiling water	20.40 ± 0.22 ^b^	<LOQ	1.79 ± 0.05 ^a,b^
	boiling 10% (*w*/*v*) NaCl	21.03 ± 0.15 ^b^	<LOQ	1.83 ± 0.05 ^a,b^
Shaking in a laboratory shaker	50% (*v*/*v*) ethanol	16.07 ± 0.08 ^a^	<LOQ	1.89 ± 0.06 ^a,b^
	80% (*v*/*v*) methanol	17.55 ± 0.11 ^a^	<LOQ	1.52 ± 0.11 ^a^
	boiling water	18.91 ± 0.22 ^a^	<LOQ	1.80 ± 0.04 ^a,b^
	boiling 10% (*w*/*v*) NaCl	20.62 ± 0.66 ^b^	<LOQ	1.56 ± 0.02 ^a^
Ultrasound-assisted extraction	50% (*v*/*v*) ethanol	16.49 ± 0.14 ^a^	<LOQ	1.05 ± 0.02 ^a,c^
	80% (*v*/*v*) methanol	17.75 ± 0.34 ^a^	<LOQ	1.53 ± 0.18 ^a^
	boiling water	19.98 ± 0.22 ^b^	<LOQ	1.87 ± 0.14 ^a,b^
	boiling 10% (*w*/*v*) NaCl	20.76 ± 0.54 ^b^	<LOQ	1.77 ± 0.05 ^a,b^

The results are presented as the mean of three independent extractions ± the SD (standard deviation). Statistically significant differences, marked by different letters, were determined using a two-way ANOVA (*p* < 0.05), followed by Tukey’s test (the effects of the solvent and extraction method were analysed separately for the sweet clover species as well as for dried and fresh plant material). <LOQ—under the limit of quantification.

**Table 2:** Content of coumarins and phenolic acids in flower extracts of *Melilotus albus* and *Melilotus officinalis*.

Sample No.		Coumarin [mg/g]	Umbelliferone [mg/g]	Melilotic Acid [mg/g]	*o*-Coumaric Acid [mg/g]	*o*-Coumaric Acid Glycoside [mg/g]	*p*-Coumaric Acid [mg/g]
***M. albus***							
1	dried	9.64 ± 0.00	0.15 ± 0.03	1.55 ± 0.02	0.40 ± 0.00	5.02 ± 0.11	0.29 ± 0.00
	fresh	23.66 ± 2.58	<LOQ	1.38 ± 0.04	<LOQ	6.04 ± 0.19	0.19 ± 0.03
2	dried	9.74 ± 0.10	0.13 ± 0.00	2.58 ± 0.02	0.36 ± 0.00	5.06 ± 0.06	0.20 ± 0.01
	fresh	24.89 ± 0.68	<LOQ	1.26 ± 0.03	<LOQ	5.13 ± 0.05	0.11 ± 0.01
3	dried	7.71 ± 0.01	0.18 ± 0.00	2.89 ± 0.04	0.43 ± 0.00	3.46 ± 0.02	0.16 ± 0.00
	fresh	19.56 ± 0.10	<LOQ	1.77 ± 0.01	<LOQ	4.07 ± 0.01	0.08 ± 0.01
4	dried	8.54 ± 0.01	0.19 ± 0.02	3.24 ± 0.11	0.35 ± 0.01	3.72 ± 0.02	0.14 ± 0.01
	fresh	25.05 ± 0.04	<LOQ	1.41 ± 0.09	<LOQ	4.67 ± 0.01	0.13 ± 0.01
5	dried	9.32 ± 0.06	0.16 ± 0.02	3.32 ± 0.02	0.29 ± 0.05	4.01 ± 0.02	0.10 ± 0.00
	fresh	24.76 ± 0.06	<LOQ	2.86 ± 0.06	<LOQ	4.23 ± 0.01	0.18 ± 0.03
6	dried	5.31 ± 0.05	0.13 ± 0.05	2.39 ± 0.02	0.27 ± 0.00	5.98 ± 0.05	0.17 ± 0.08
	fresh	12.36 ± 0.05	<LOQ	1.55 ± 0.17	<LOQ	5.95 ± 0.06	0.09 ± 0.01
7	dried	5.85 ± 0.02	0.19 ± 0.00	2.41 ± 0.11	0.21 ± 0.00	4.31 ± 0.03	0.10 ± 0.00
	fresh	19.68 ± 0.89	<LOQ	0.84 ± 0.00	<LOQ	4.04 ± 0.06	0.13 ± 0.00
***M. officinalis***							
8	dried	8.24 ± 0.14	0.10 ± 0.01	2.28 ± 0.00	0.98 ± 0.00	3.46 ± 0.03	0.08 ± 0.01
	fresh	26.30 ± 0.17	<LOQ	2.18 ± 0.02	<LOQ	3.67 ± 0.01	0.06 ± 0.00
9	dried	5.84 ± 0.01	0.15 ± 0.01	3.97 ± 0.00	1.11 ± 0.00	3.18 ± 0.08	0.13 ± 0.03
	fresh	11.31 ± 0.05	<LOQ	1.60 ± 0.01	<LOQ	3.73 ± 0.03	0.06 ± 0.00
10	dried	7.95 ± 0.05	0.11 ± 0.01	2.94 ± 0.02	0.90 ± 0.00	6.01 ± 0.00	0.14 ± 0.00
	fresh	18.30 ± 0.02	<LOQ	2.20 ± 0.01	<LOQ	5.25 ± 0.08	0.09 ± 0.00

Results are presented as means from three independent extractions ± SD (standard deviation); <LOQ—under limit of quantification. Numbers 1–7 represent sweet clover plants collected from various locations in Podkarpackie Voivodeship, Poland.

**Table 3:** Content of flavonoids in flower extracts of *Melilotus albus* and *Melilotus officinalis*.

Sample No.		Hyperoside [mg/g]	Quercetin [mg/g]	Quercetin Glycoside [mg/g]	Luteolin [mg/g]	Kaempferol Glycoside [mg/g]
***M. albus***						
1	dried	14.88 ± 0.01	1.21 ± 0.01	2.77 ± 0.00	0.85 ± 0.02	<LOD
	fresh	16.94 ± 0.57	1.15 ± 0.01	1.32 ± 0.04	0.42 ± 0.01	<LOD
2	dried	14.77 ± 0.14	1.53 ± 0.22	2.78 ± 0.03	1.31 ± 0.00	<LOD
	fresh	15.49 ± 0.60	1.46 ± 0.05	1.44 ± 0.03	0.79 ± 0.05	<LOD
3	dried	13.83 ± 0.03	1.28 ± 0.02	1.96 ± 0.01	1.11 ± 0.14	<LOD
	fresh	14.33 ± 0.02	1.14 ± 0.05	0.65 ± 0.08	0.75 ± 0.00	<LOD
4	dried	14.77 ± 0.06	0.88 ± 0.00	1.80 ± 0.00	1.00 ± 0.96	<LOD
	fresh	17.38 ± 0.03	0.76 ± 0.19	0.46 ± 0.01	0.65 ± 0.01	<LOD
5	dried	14.23 ± 0.01	1.71 ± 0.05	1.90 ± 0.04	0.77 ± 0.00	<LOD
	fresh	15.77 ± 0.20	1.25 ± 0.20	0.52 ± 0.00	0.41 ± 0.00	<LOD
6	dried	10.58 ± 0.30	1.44 ± 0.01	3.06 ± 0.17	0.69 ± 0.01	<LOD
	fresh	13.70 ± 0.01	1.09 ± 0.01	1.69 ± 0.00	0.42 ± 0.01	<LOD
7	dried	20.36 ± 0.30	1.37 ± 0.22	3.11 ± 0.04	0.53 ± 0.01	<LOD
	fresh	19.02 ± 0.50	1.38 ± 0.06	1.95 ± 0.05	0.49 ± 0.02	<LOD
***M. officinalis***						
8	dried	2.20 ± 0.05	1.47 ± 0.03	3.58 ± 0.06	0.74 ± 0.01	1.39 ± 0.91
	fresh	3.02 ± 0.04	1.50 ± 0.00	1.45 ± 0.06	0.57 ± 0.01	0.10 ± 0.00
9	dried	2.19 ± 0.30	1.71 ± 0.06	2.61 ± 0.22	1.09 ± 0.24	1.03 ± 0.00
	fresh	1.44 ± 0.01	1.30 ± 0.27	1.63 ± 0.02	0.73 ± 0.00	0.31 ± 0.00
10	dried	2.81 ± 0.00	1.62 ± 0.02	2.53 ± 0.05	0.88 ± 0.00	1.32 ± 0.02
	fresh	2.07 ± 0.02	1.52 ± 0.10	1.46 ± 0.03	0.78 ± 0.01	0.09 ± 0.00

Results are presented as means from three independent extractions ± SD (standard deviation); <LOD—under limit of detection. Numbers 1–7 represent sweet clover plants collected from various locations in Podkarpackie Voivodeship, Poland.

**Table 4:** Content of coumarins and phenolic acids in leaf extracts of *Melilotus albus* and *Melilotus officinalis*.

Sample No.		Coumarin [mg/g]	Umbelliferone [mg/g]	Melilotic Acid [mg/g]	*o*-Coumaric Acid [mg/g]	*o*-Coumaric Acid Glycoside [mg/g]	*p*-Coumaric Acid [mg/g]
***M. albus***							
1	dried	3.24 ± 0.03	0.37 ± 0.02	4.76 ± 0.05	0.08 ± 0.01	1.17 ± 0.01	0.05 ± 0.00
	fresh	11.65 ± 0.08	0.26 ± 0.01	14.93 ± 0.37	<LOQ	2.09 ± 0.02	<LOQ
2	dried	1.60 ± 0.02	0.59 ± 0.05	6.35 ± 0.05	0.07 ± 0.000	1.26 ± 0.02	0.05 ± 0.01
	fresh	6.60 ± 0.18	0.46 ± 0.03	19.76 ± 0.11	<LOQ	1.07 ± 0.02	<LOQ
3	dried	1.78 ± 0.00	0.61 ± 0.07	9.22 ± 0.04	0.13 ± 0.00	1.38 ± 0.00	0.04 ± 0.01
	fresh	7.25 ± 0.01	0.37 ± 0.01	13.13 ± 0.25	<LOQ	1.96 ± 0.03	<LOQ
4	dried	1.30 ± 0.05	0.41 ± 0.04	8.17 ± 0.05	0.11 ± 0.00	1.36 ± 0.02	0.05 ± 0.00
	fresh	9.86 ± 0.17	0.45 ± 0.05	13.54 ± 0.18	<LOQ	1.25 ± 0.03	<LOQ
5	dried	1.28 ± 0.06	0.23 ± 0.01	6.53 ± 0.31	0.08 ± 0.00	1.09 ± 0.11	0.05 ± 0.00
	fresh	9.77 ± 0.16	0.26 ± 0.00	11.65 ± 0.03	<LOQ	1.34 ± 0.02	<LOQ
6	dried	1.62 ± 0.01	0.27 ± 0.00	3.60 ± 0.01	0.17 ± 0.00	2.01 ± 0.00	0.06 ± 0.00
	fresh	9.57 ± 0.02	0.38 ± 0.00	6.27 ± 0.01	<LOQ	2.96 ± 0.03	<LOQ
7	dried	0.80 ± 0.01	0.51 ± 0.04	1.23 ± 0.00	0.02 ± 0.00	0.61 ± 0.00	0.03 ± 0.00
	fresh	6.76 ± 0.08	0.44 ± 0.05	2.32 ± 0.01	<LOQ	0.49 ± 0.00	<LOQ
***M. officinalis***							
8	dried	1.36 ± 0.01	0.52 ± 0.01	3.60 ± 0.00	0.05 ± 0.00	0.67 ± 0.00	0.04 ± 0.00
	fresh	7.33 ± 0.02	0.51 ± 0.00	7.03 ± 0.00	<LOQ	1.36 ± 0.00	<LOQ
9	dried	1.34 ± 0.01	0.23 ± 0.01	9.21 ± 0.02	0.21 ± 0.00	1.10 ± 0.11	0.03 ± 0.00
	fresh	6.44 ± 0.12	0.43 ± 0.02	9.31 ± 0.17	<LOQ	0.99 ± 0.02	<LOQ
10	dried	1.95 ± 0.02	0.27 ± 0.00	3.68 ± 0.01	0.42 ± 0.00	0.58 ± 0.00	0.05 ± 0.00
	fresh	9.83 ± 0.01	0.40 ± 0.01	8.06 ± 0.02	<LOQ	1.30 ± 0.02	<LOQ

Results are presented as means from three independent extractions ± SD (standard deviation); <LOQ—under limit of quantification. Numbers 1–7 represent sweet clover plants collected from various locations in Podkarpackie Voivodeship, Poland.

**Table 5:** Content of flavonoids in leaf extracts of *Melilotus albus* and *Melilotus officinalis*.

Sample No.		Hyperoside [mg/g]	Quercetin [mg/g]	Quercetin Glycoside [mg/g]	Luteolin [mg/g]	Kaempferol Glycoside [mg/g]
***M. albus***						
1	dried	3.84 ± 0.05	0.12 ± 0.02	0.39 ± 0.00	0.15 ± 0.01	<LOD
	fresh	4.75 ± 0.11	0.10 ± 0.02	0.35 ± 0.01	0.11 ± 0.00	<LOD
2	dried	7.15 ± 0.07	0.10 ± 0.00	0.55 ± 0.01	0.10 ± 0.00	<LOD
	fresh	5.01 ± 0.11	0.07 ± 0.00	0.46 ± 0.01	0.07 ± 0.00	<LOD
3	dried	3.47 ± 0.01	0.14 ± 0.00	0.28 ± 0.02	0.13 ± 0.02	<LOD
	fresh	5.34 ± 0.06	0.20 ± 0.04	0.20 ± 0.01	0.12 ± 0.01	<LOD
4	dried	3.85 ± 0.08	0.20 ± 0.02	0.32 ± 0.00	0.16 ± 0.00	<LOD
	fresh	2.78 ± 0.15	0.23 ± 0.05	0.20 ± 0.01	0.14 ± 0.01	<LOD
5	dried	3.15 ± 0.02	0.10 ± 0.01	0.23 ± 0.02	0.15 ± 0.02	<LOD
	fresh	2.42 ± 0.04	0.10 ± 0.03	0.09 ± 0.00	0.08 ± 0.00	<LOD
6	dried	5.49 ± 0.00	0.06 ± 0.00	0.57 ± 0.02	0.09 ± 0.00	<LOD
	fresh	4.49 ± 0.00	0.07 ± 0.00	0.38 ± 0.01	0.07 ± 0.00	<LOD
7	dried	2.93 ± 0.02	0.18 ± 0.00	0.22 ± 0.01	0.09 ± 0.01	<LOD
	fresh	2.43 ± 0.06	0.04 ± 0.01	0.17 ± 0.03	0.08 ± 0.03	<LOD
***M. officinalis***						
8	dried	2.02 ± 0.01	0.10 ± 0.00	0.71 ± 0.00	0.16 ± 0.02	0.11 ± 0.00
	fresh	2.86 ± 0.01	0.08 ± 0.01	0.67 ± 0.01	0.13 ± 0.00	0.07 ± 0.00
9	dried	3.15 ± 0.02	0.26 ± 0.01	0.45 ± 0.02	0.12 ± 0.01	0.10 ± 0.00
	fresh	2.53 ± 0.05	0.14 ± 0.00	0.48 ± 0.01	0.10 ± 0.00	0.07 ± 0.00
10	dried	4.82 ± 0.00	0.09 ± 0.00	0.59 ± 0.01	0.08 ± 0.00	0.11 ± 0.00
	fresh	2.03 ± 0.00	0.07 ± 0.01	0.52 ± 0.01	0.05 ± 0.00	0.05 ± 0.01

Results are presented as means from three independent extractions ± SD (standard deviation); <LOD—under limit of detection. Numbers 1–7 represent sweet clover plants collected from various locations in Podkarpackie Voivodeship, Poland.

**Table 6:** Antioxidant activity and total phenolic content of tested flower and leaf extracts of *Melilotus albus* and *Melilotus officinalis*.

Sample No.	FRAP [µmol TE/g]	DPPH· [µmol TE/g]	TPC [mg GAE/g]
***M. albus*** **flowers**			
1	59.30 ± 2.75	56.12 ± 3.40	20.41 ± 0.89
2	56.62 ± 1.33	61.54 ± 5.02	24.53 ± 1.81
3	46.85 ± 0.45	43.06 ± 3.01	20.43 ± 0.16
4	43.54 ± 1.30	40.31 ± 0.13	22.27 ± 0.48
5	55.99 ± 1.63	41.55 ± 2.63	21.49 ± 1.21
6	57.88 ± 4.16	50.61 ± 0.38	21.78 ± 0.73
7	56.20 ± 3.27	51.06 ± 2.00	23.83 ± 1.97
***M. officinalis*** **flowers**			
8	82.62 ± 1.11	73.53 ± 0.12	31.85 ± 0.38
9	82.41 ± 1.26	73.53 ± 0.13	35.41 ± 0.38
10	83.14 ± 0.22	72.73 ± 4.51	30.43 ± 0.16
***M. albus*** **leaves**			
1	75.95 ± 0.15	117.48 ± 0.33	33.02 ± 0.83
2	67.12 ± 0.02	103.21 ± 0.34	26.13 ± 0.12
3	69.75 ± 0.30	114.86 ± 0.67	29.82 ± 0.00
4	75.42 ± 2.38	138.41 ± 0.34	31.89 ± 0.38
5	69.22 ± 2.82	102.25 ± 1.68	29.14 ± 1.47
6	74.47 ± 3.38	123.66 ± 2.65	30.72 ± 1.78
7	71.85 ± 0.59	104.87 ± 2.69	22.79 ± 0.25
***M. officinalis*** **leaves**			
8	88.24 ± 5.05	173.61 ± 1.00	44.82 ± 0.06
9	86.55 ± 0.89	171.00 ± 5.70	39.19 ± 3.69
10	99.68 ± 4.90	188.36 ± 1.00	42.57 ± 0.96

The numbers 1–7 represent sweet clover plants collected from various locations in the Podkarpackie Voivodeship, Poland.

**Table 7:** Characteristics of the plant material collected from the Podkarpackie Voivodeship.

Sample No.	Species	Voucher Number	Location	Altitude [m]	Year
1	*Melilotus albus*	2022/07/MAB1	Borówki (49°57′07″ N, 22°09′37″ E)	353.41	2022
2	*Melilotus albus*	2022/07MAB2	Borówki (49°57′07″ N, 22°09′37″ E)	353.34	2022
3	*Melilotus albus*	2022/07/MAS1	Sonina (50°03′44″ N, 22°16′12″ E)	196.73	2022
4	*Melilotus albus*	2022/07/MAS2	Sietesz (49°59′16″ N, 22°20′45″ E)	263.18	2022
5	*Melilotus albus*	2022/07/MAR1	Rzeszów (49°58′54″ N, 21°57′41″ E)	206.66	2022
6	*Melilotus albus*	2021/07/MAC1	Chmielnik (49°58′43″ N, 22°08′01″ E)	251.00	2021
7	*Melilotus albus*	2022/07/MAWR1	Wola Rzeczycka (50°38′27″ N, 22°01′54″ E)	150.28	2022
8	*Melilotus officinalis*	2022/07/MOB1	Borówki (49°57′07″ N, 22°09′37″ E)	353.32	2022
9	*Melilotus officinalis*	2022/07/MOR1	Rzeszów (49°58′54″ N, 21°57′41″ E)	206.54	2022
10	*Melilotus officinalis*	2021/07/MOC1	Chmielnik (49°58′43″ N, 22°08′01″ E)	251.27	2021

## Data Availability

The data presented in this study are available in the article.

## Supplementary Materials

The following supporting information can be downloaded at https://www.mdpi.com/article/10.3390/molecules30030526/s1: Table S1: Content of coumarins and phenolic acids in stem extracts of *Melilotus albus* and *Melilotus officinalis*; Table S2: Content of flavonoids in stem extracts of *Melilotus albus* and *Melilotus officinalis*; Table S3: Validation parameters for HPLC method; Figure S1: HPLC-PDA chromatogram of phenolic compounds of dried *Melilotus albus* flower extracts recorded at 280 nm.
