# Supplementary material for: Sweet Clover (Melilotus spp.) as a Source of Biologically Active Compounds
Source: Molecules. 2025 Jan 24;30(3):526. doi: 10.3390/molecules30030526 (PMC11820964; doi:10.3390/molecules30030526)
Supplement: Supplementary file 1 [file molecules-30-00526-s001.zip › molecules-3366053-supplementary.pdf]

molecules

*Article*

# Sweet clover (*Melilotus* spp.) as a source of biologically active compounds

**Patrycja Sowa-Borowiec [1], * Maria Czernicka [2], Wacław Jarecki [3,*] and Małgorzata Dżugan [4]**

[1] Department of General and Inorganic Chemistry, Faculty of Chemical Engineering and Technology, Cracow University of Technology, 31-155 Cracow, Poland

[2] Department of Bioenergetics, Food Analysis and Microbiology, University of Rzeszow, 35-601 Rzeszow, Poland; mczernicka@ur.edu.pl

[3] Department of Crop Production, University of Rzeszów, Zelwerowicza 4, 35-601 Rzeszow, Poland

[4] Department of Chemistry and Food Toxicology, University of Rzeszów, Ćwiklińskiej 1a, 35-601 Rzeszow, Poland

* Correspondence: patrycja.sowa-borowiec@pk.edu.pl; wjarecki@ur.edu.pl

 https://doi.org/10.3390/molecules30030526

**Tabel S1.** Content of coumarins and phenolic acids in stem extracts of *Melilotus albus* and *Melilotus officinalis*.

| Sample No. | | Coumarin [mg/g] | Umbelliferone [mg/g] | Melilotic acid [mg/g] | *o*-coumaric acid [mg/g] | *o*-coumaric acid glycoside [mg/g] | *p*-coumaric acid [mg/g] |
|---|---|---|---|---|---|---|---|
| | | | | ***M. albus*** | | | |
| 1 | dried | 0.75 ± 0.00 | 0.07 ± 0.00 | 0.51 ± 0.00 | 0.01 ± 0.00 | 0.30 ± 0.00 | 0.01 ± 0.00 |
| | fresh | 4.97 ± 0.01 | 0.06 ± 0.00 | 0.45 ± 0.04 | <LOQ | 0.33 ± 0.00 | <LOQ |
| 2 | dried | 0.68 ± 0.01 | 0.11 ± 0.01 | 0.71 ± 0.00 | 0.02 ± 0.00 | 0.38 ± 0.01 | 0.01 ± 0.00 |
| | fresh | 3.60 ± 0.03 | 0.10 ± 0.00 | 0.57 ± 0.00 | <LOQ | 0.35 ± 0.01 | <LOQ |
| 3 | dried | 1.06 ± 0.00 | 0.08 ± 0.00 | 1.30 ± 0.01 | 0.03 ± 0.00 | 0.20 ± 0.00 | 0.01 ± 0.00 |
| | fresh | 4.58 ± 0.02 | 0.07 ± 0.00 | 0.95 ± 0.02 | <LOQ | 0.19 ± 0.00 | <LOQ |
| 4 | dried | 0.85 ± 0.01 | 0.11 ± 0.01 | 1.28 ± 0.02 | 0.02 ± 0.00 | 0.21 ± 0.00 | 0.02 ± 0.00 |
| | fresh | 5.97 ± 0.02 | 0.11 ± 0.00 | 0.94 ± 0.01 | <LOQ | 0.26 ± 0.00 | <LOQ |
| 5 | dried | 0.59 ± 0.01 | 0.07 ± 0.00 | 0.43 ± 0.01 | 0.01 ± 0.00 | 0.07 ± 0.00 | 0.03 ± 0.00 |
| | fresh | 4.40 ± 0.01 | 0.19 ± 0.01 | 0.29 ± 0.00 | <LOQ | 0.14 ± 0.00 | <LOQ |
| 6 | dried | 0.70 ± 0.00 | 0.07 ± 0.01 | 0.92 ± 0.00 | 0.01 ± 0.00 | 0.31 ± 0.00 | 0.02 ± 0.00 |
| | fresh | 3.27 ± 0.00 | 0.07 ± 0.00 | 0.71 ± 0.01 | <LOQ | 0.38 ± 0.00 | <LOQ |
| 7 | dried | 0.66 ± 0.02 | 0.14 ± 0.01 | 0.66 ± 0.03 | 0.01 ± 0.00 | 0.30 ± 0.01 | 0.01 ± 0.00 |
| | fresh | 4.13 ± 0.01 | 0.13 ± 0.00 | 0.46 ± 0.00 | <LOQ | 0.27 ± 0.00 | <LOQ |
| | | | | ***M. officinalis*** | | | |
| 8 | dried | 0.55 ± 0.01 | 0.07 ± 0.00 | 0.30 ± 0.01 | 0.01 ± 0.00 | 0.14 ± 0.00 | 0.02 ± 0.00 |
| | fresh | 3.06 ± 0.02 | 0.05 ± 0.00 | 0.17 ± 0.01 | <LOQ | 0.15 ± 0.00 | <LOQ |
| 9 | dried | 0.83 ± 0.02 | 0.05 ± 0.00 | 2.79 ± 0.04 | 0.04 ± 0.00 | 0.18 ± 0.00 | 0.02 ± 0.00 |
| | fresh | 4.40 ± 0.00 | 0.05 ± 0.01 | 1.18 ± 0.02 | <LOQ | 0.19 ± 0.01 | <LOQ |
| 10 | dried | 0.66 ± 0.01 | 0.20 ± 0.00 | 1.51 ± 0.00 | 0.01 ± 0.00 | 0.17 ± 0.00 | 0.02 ± 0.00 |
| | fresh | 2.36 ± 0.01 | 0.22 ± 0.01 | 0.95 ± 0.00 | <LOQ | 0.13 ± 0.00 | <LOQ |

Results are presented as means from three independent extractions ± SD (standard deviation); <LOD – under limit of detection. Numbers 1–7 represent sweet clover plants collected from various locations in the Podkarpackie Voivodeship, Poland.

**Table S2.** Content of flavonoids in stem extracts of *Melilotus albus* and *Melilotus officinalis*.

| Samples No. | | Hyperoside [mg/g] | Quercetin [mg/g] | Quercetin glycoside [mg/g] | Luteolin [mg/g ] | Kaempferol glycoside [mg/g] |
|---|---|---|---|---|---|---|
| | | | | ***M. albus*** | | |
| 1 | dried | 1.26 ± 0.00 | 0.02 ± 0.00 | 0.40 ± 0.00 | 0.11 ± 0.00 | <LOD |
| | fresh | 1.21 ± 0.00 | <LOQ | 0.33 ± 0.00 | 0.12 ± 0.00 | <LOD |
| 2 | dried | 1.36 ± 0.02 | 0.03 ± 0.00 | 0.52 ± 0.01 | 0.09 ± 0.00 | <LOD |
| | fresh | 1.37 ± 0.08 | <LOQ | 0.19 ± 0.00 | 0.08 ± 0.00 | <LOD |
| 3 | dried | 0.97 ± 0.01 | 0.05 ± 0.00 | 0.25 ± 0.00 | 0.05 ± 0.02 | <LOD |
| | fresh | 1.03 ± 0.00 | <LOQ | 0.10 ± 0.00 | 0.05 ± 0.02 | <LOD |
| 4 | dried | 0.86 ± 0.00 | 0.04 ± 0.00 | 0.17 ± 0.00 | 0.03 ± 0.00 | <LOD |
| | fresh | 1.15 ± 0.00 | <LOQ | 0.13 ± 0.00 | 0.06 ± 0.00 | <LOD |
| 5 | dried | 0.57 ± 0.02 | 0.02 ± 0.00 | 0.30 ± 0.00 | 0.04 ± 0.00 | <LOD |
| | fresh | 0.82 ± 0.02 | <LOQ | 0.20 ± 0.00 | 0.07 ± 0.00 | <LOD |
| 6 | dried | 1.32 ± 0.01 | 0.03 ± 0.00 | 0.33 ± 0.00 | 0.10 ± 0.00 | <LOD |
| | fresh | 1.69 ± 0.01 | <LOQ | 0.16 ± 0.00 | 0.11 ± 0.01 | <LOD |
| 7 | dried | 1.33 ± 0.03 | 0.03 ± 0.00 | 0.47 ± 0.01 | 0.02 ± 0.00 | <LOD |
| | fresh | 1.70 ± 0.02 | <LOQ | 0.13 ± 0.00 | 0.06 ± 0.01 | <LOD |
| | | | | ***M. officinalis*** | | |
| 8 | dried | 0.29 ± 0.01 | 0.05 ± 0.00 | 0.33 ± 0.00 | 0.07 ± 0.00 | 0.21 ± 0.00 |
| | fresh | 0.34 ± 0.00 | <LOQ | 0.15 ± 0.00 | 0.11 ± 0.00 | 0.02 ± 0.00 |
| 9 | dried | 0.63 ± 0.02 | 0.04 ± 0.00 | 0.29 ± 0.00 | 0.06 ± 0.00 | 0.06 ± 0.00 |
| | fresh | 0.53 ± 0.01 | <LOQ | 0.20 ± 0.00 | 0.07 ± 0.00 | 0.03 ± 0.00 |
| 10 | dried | 0.45 ± 0.01 | 0.04 ± 0.00 | 0.32 ± 0.00 | 0.07 ± 0.00 | 0.07 ± 0.00 |
| | fresh | 0.40 ± 0.00 | <LOQ | 0.30 ± 0.01 | 0.10 ± 0.01 | 0.03 ± 0.00 |

Results are presented as means from three independent extractions ± SD (standard deviation); <LOD – under limit of detection. Numbers 1–7 represent sweet clover plants collected from various locations in the Podkarpackie Voivodeship, Poland.

**Table S3**. Validation Parameters for HPLC Method.

| Compound | Linearity range [μg/ml] | $R^2$ | Calibration curve | LOD [μg/ml] | LOQ [μg/ml] | % RSD | |
|---|---|---|---|---|---|---|---|
| | | | | | | Intra-day | Inter-day |
| coumarin | 5-100 | 0.9999 | $y = 86841x - 70.092$ | 0.06 | 0.10 | 0.50 | 1.20 |
| *o*-coumaric acid | 5-100 | 0.9999 | $y = 103602x + 38.72$ | 0.09 | 0.20 | 0.70 | 1.20 |
| p-coumaric acid | 5-50 | 0.9999 | $y = 93898x - 20.37$ | 0.06 | 0.40 | 1.40 | 2.60 |
| melilotic acid | 5-100 | 0.9985 | $y = 9594.1x - 11.46$ | 0.10 | 0.20 | 0.50 | 2.10 |
| quercetin | 5-100 | 0.9951 | $y = 50136x + 36.36$ | 0.30 | 0.60 | 0.60 | 0.90 |
| hyperoside | 5-100 | 0.9996 | $y = 116421x - 99.42$ | 0.30 | 0.60 | 0.50 | 1.60 |
| umbelliferone | 5-100 | 0.9981 | $y = 58496x + 32.64$ | 0.10 | 0.30 | 0.20 | 1.50 |
| luteolin | 5-100 | 0.9964 | $y = 94579x - 30.24$ | 0.09 | 0.20 | 0.70 | 1.60 |

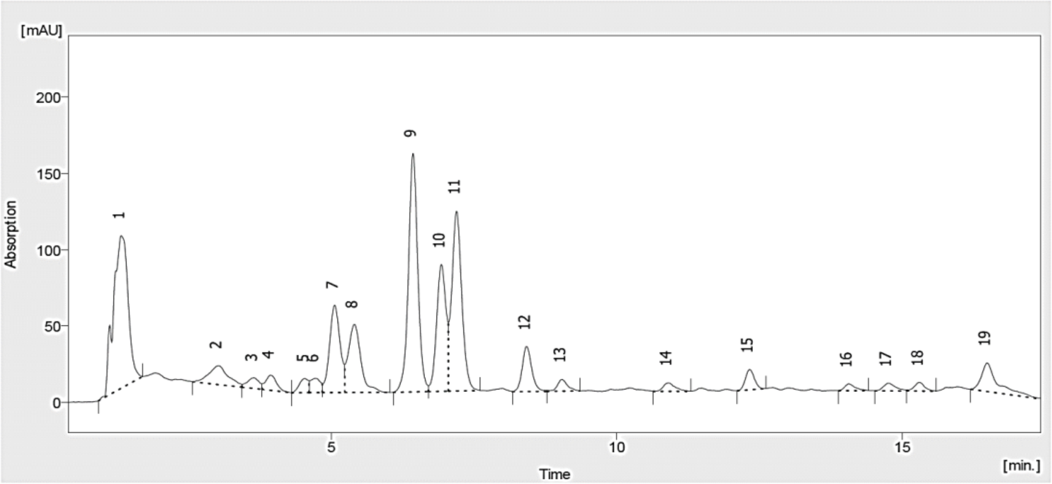


**Figure S1.** HPLC- PDA chromatogram of phenolic compounds of *Melilotus albus* dried flower extracts recorded at 280 nm (1– un dentified compound, 2– *o*-coumaric acid glycoside, 3– unidentified compound, 4– *p*-coumaric acid, 7– melilotic acid, 8– umbellifer-one, 9– coumarin, 10– *o*-coumaric acid, 11– hyperoside, 12– quercetin glycoside, 15– quercetin, 19– luteolin, 13, 14, 16, 17, 18– flavonol derivatives).
